# Treatment of Major Depressive Disorder with Iyengar Yoga and Coherent Breathing: A Randomized Controlled Dosing Study

**DOI:** 10.1089/acm.2016.0140

**Published:** 2017-03-01

**Authors:** Chris C. Streeter, Patricia L. Gerbarg, Theodore H. Whitfield, Liz Owen, Jennifer Johnston, Marisa M. Silveri, Marysia Gensler, Carol L. Faulkner, Cathy Mann, Mary Wixted, Anne Marie Hernon, Maren B. Nyer, E. Richard P. Brown, John E. Jensen

**Affiliations:** ^1^ Department of Psychiatry, Boston University School of Medicine, Boston, MA.; ^2^ Department of Neurology, Boston University School of Medicine, Boston, MA.; ^3^ Department of Psychiatry, Harvard School of Medicine, Boston, MA.; ^4^ Department of Psychiatry, Boston Medical Center, Boston, MA.; ^5^ Department of Psychiatry, McLean Hospital, Belmont, MA.; ^6^ Department of Psychiatry, Edith Nourse Rogers Memorial Veterans Hospital, Bedford, MA.; ^7^ Department of Psychiatry, New York Medical College, Valhalla, NY.; ^8^ Biostatistical Solutions, Boston, MA.; ^9^ Department of Psychiatry, Massachusetts General Hospital, Boston MA; ^10^ Department of Psychiatry, Columbia University College of Physicians and Surgeons, New York, NY.

**Keywords:** yoga, depression, Iyengar, breathing, randomized, dosing

## Abstract

***Objectives:*** The aims of this study were to assess the effects of an intervention of Iyengar yoga and coherent breathing at five breaths per minute on depressive symptoms and to determine optimal intervention yoga dosing for future studies in individuals with major depressive disorder (MDD).

***Methods:*** Subjects were randomized to the high-dose group (HDG) or low-dose group (LDG) for a 12-week intervention of three or two intervention classes per week, respectively. Eligible subjects were 18–64 years old with MDD, had baseline Beck Depression Inventory-II (BDI-II) scores ≥14, and were either on no antidepressant medications or on a stable dose of antidepressants for ≥3 months. The intervention included 90-min classes plus homework. Outcome measures were BDI-II scores and intervention compliance.

***Results:*** Fifteen HDG (*M*_age_ = 38.4 ± 15.1 years) and 15 LDG (*M*_age_ = 34.7 ± 10.4 years) subjects completed the intervention. BDI-II scores at screening and compliance did not differ between groups (*p* = 0.26). BDI-II scores declined significantly from screening (24.6 ± 1.7) to week 12 (6.0 ± 3.8) for the HDG (–18.6 ± 6.6; *p* < 0.001), and from screening (27.7 ± 2.1) to week 12 (10.1 ± 7.9) in the LDG (–17.7 ± 9.3; *p* < 0.001). There were no significant differences between groups, based on response (i.e., >50% decrease in BDI-II scores; *p* = 0.65) for the HDG (13/15 subjects) and LDG (11/15 subjects) or remission (i.e., number of subjects with BDI-II scores <14; *p* = 1.00) for the HDG (14/15 subjects) and LDG (13/15 subjects) after the 12-week intervention, although a greater number of subjects in the HDG had 12-week BDI-II scores ≤10 (*p* = 0.04).

***Conclusion:*** During this 12-week intervention of yoga plus coherent breathing, depressive symptoms declined significantly in patients with MDD in both the HDG and LDG. Both groups showed comparable compliance and clinical improvements, with more subjects in the HDG exhibiting BDI-II scores ≤10 at week 12.

## Introduction

Major depressive disorder (MDD) is common, recurrent, chronic, and disabling. Due in part to its prevalence, depression is globally responsible for more years lost to disability than any other disease.^1^ Up to 50% of individuals treated with antidepressant medications for MDD do not achieve full remission.^2^ Thus, the currently available treatments for depression do not effectively or sufficiently reduce the associated morbidity or mortality.^3^ To reduce the burden of MDD, more effective and adjunctive therapies are needed.

Yoga-based therapies offer promise as both monotherapies and adjunctive treatments. A meta-analysis and a review of randomized controlled trials (RCTs) using yoga as a treatment for depression found that yoga was significantly better than usual care, relaxation exercises, and aerobic exercise.^4,5^ One RTC that demonstrated positive results employed Iyengar yoga, and assessed symptom reduction using the Beck Depression Inventory, similar to the intervention and assessment used in this study.^6^

In disorders with low parasympathetic tone (as measured by low heart-rate variability [HRV]), such as MDD, yoga-based interventions are associated with symptomatic improvement.^7^ Slow breathing exercises have also been linked to increased HRV and improved mood.^8^ In subjects with MDD treated with resonant breathing (coherent breathing with pursed lips resistive exhalation) at 4.5–6.5 breaths per minute (bpm), HRV increased and mood improved.^9^

Therefore, the objectives of this study were to assess the effects of an intervention combining Iyengar yoga and coherent breathing on depressive symptoms in subjects with MDD, and to determine the optimal dose of the yoga intervention for future RTCs.

## Materials and Methods

### Instruments and data management

This study was approved by the Boston University Medical Center (BUMC) Institutional Review Board. Written informed consent was obtained from all participants during the screening interview. The screening and intervention occurred at BUMC from October 2013 to September 2015. Subjects were recruited from the Internet and local advertisements. The randomization code was generated using a permuted block design (*n* = 4). The randomization numbers and group assignments were kept in sealed envelopes, numbered sequentially, and opened in sequence when a subject was randomized. Mood scales were self-completed by subjects, such that unblinded research staff did not administer mood scales. The Structured Clinical Interview for DSM-IV Axis I Disorders (SCID) was used to diagnose presence of Axis I Disorders.^10^ The Beck Depression Inventory-II (BDI-II), a 21-item, self-completed questionnaire, was used to track depressive symptoms with the following scoring: severe depression 29–63, moderate depression 20–28, mild depression 14–19, and minimal depression 0–13.^11^ Other mood scales completed will be reported elsewhere. The Columbia-Suicide Severity Rating Scale (C-SSRS) was used to assess suicide risk.^12^ Timeline Follow Back was used to determine alcohol consumption for the 3 months prior to screening.^13^ Study data were collected using Research Electronic Data Capture (REDCap).^14^

### Inclusion and exclusion criteria

The study began with the following inclusion criteria: 18–55 years old; current diagnosis of MDD; BDI-II score ≥14 and <28 at screening; comorbid anxiety disorder(s) were allowable if they would not interfere with participation. The following were exclusionary: treatment with antidepressants, benzodiazepines, or mood stabilizers prior to screening; psychotherapy for depression in the 3 months prior to screening; more than six 1 h mind–body practices in the last 6 months; a current prayer practice >2 h a week; bipolar illness; history of psychosis; suicide attempt throughout lifetime or suicidal ideation within the last year; current alcohol or substance abuse or dependence; and inability to complete the study protocol.

After enrolling 16/32 subjects, it was determined that the criteria were unnecessarily restrictive. Therefore, inclusion criteria were expanded: upper age limit to 65 years; BDI-II upper limit of 28 removed; subjects taking a stable dose (no change in the amount) of antidepressants for at least 3 months prior to screen if no anticipated dose changes during the study. Exclusion criteria were also expanded: suicide attempts within the prior year instead of lifetime; and to allow suicidal ideation without intent, but to exclude suicidal ideation with intent in the prior year using C-SSRS criteria.

### Yoga plus coherent breathing intervention

The manualized Iyengar protocol developed by the authors' group was modified such that the Iyengar posture protocol was followed by a period of relaxation transition to a coherent breathing exercise to enhance the potential benefit to individuals with MDD.^6^ The yoga component emphasized backbends and inversions because the Iyengar method identifies these postures as useful for treating depression.^15^ During relaxation transition, subjects performed ujjayi breathing, which creates a small resistance to airflow by slight contraction of the laryngeal muscles with partial closure of the glottis, activating vagal afferents (originating in the larynx and pharynx), while also activating vagal afferents by breathing against a resistive load.^16^ Coherent breathing entails gentle breathing through the nose with equal duration of inhalation and exhalation at a respiratory rate that optimizes HRV and sympatho-vagal balance by breathing at an average rate of 5 bpm for most adults.^8^ Data on the changes in HRV will be reported elsewhere.

The 90-min yoga classes consisted of approximately 60 min of yoga postures and approximately 10 min of transition that included deep relaxation (savasana) and ujjayi breathing (which helps control and slow the breath rate),^16^ followed by 20 min of coherent breathing. Subjects were given the option to continue ujjayi during coherent breathing. A compact disc (CD) of two chime tones was played to pace the coherent breathing with instructions to breathe in on the high tone and out on the low tone. The 12-week yoga intervention was comprised of 11 unique sequences. The first 10 sequences were done for 1 week each, and the 11th sequence extended over a 2-week period. Each homework assignment consisted of 15 min of postures and 15 min of CD-paced coherent breathing. The posture homework was guided by weeks 2, 4, and 11 of the manual. The BDI-II was completed at screening and weeks 4, 8, and 12.

All yoga classes followed an established progression: seated or reclining postures for centering while the sutra translation and interpretation for that class were read; Sun Salutations; standing poses; twists, transition poses; inversions; deep relaxation (shavasana and ujjayi); and coherent breathing. Ujjayi breathing was reviewed at the beginning of each class and during the transition period. The names of the postures in English and Sanskrit and sequences for each week are provided in Supplementary Tables 1 and 2 (Supplementary Data are available online at www.liebertpub.com/acm).

This intervention used the following props per participant: a yoga mat, two yoga blocks, a yoga belt, a bolster (6 inches × 12 inches × 24 inches), and three yoga blankets. For at home practice, each subject was given the intervention manual, a yoga mat, a 15-min CD for pacing home-breathing practice, and two yoga blocks.

To determine the optimal dose of yoga, subjects were randomized to a high-dose group (HDG) or a low-dose group (LDG) for 12 weeks. The HDG consisted of three 90-min yoga classes and four 30-min homework sessions per week. The LDG consisted of two 90-min yoga classes and three 30-min homework sessions per week. Subject compliance was monitored by sign-in sheets at each yoga class, and through weekly forms assessing compliance with homework. A rolling admissions design was employed to accommodate the scheduling requirements of imaging sessions. Subjects entered the 12-week protocol as they were randomized.

### Yoga teacher training and considerations

All yoga instructors had passed the Iyengar Introductory Level II certification exam, requiring ≥2 years of study, acquired at least 5 years teaching experience, and received training to modify yoga postures to accommodate each participant's balance, strength, and flexibility using posture modification, prop utilization, and sequence modification within the established progression of poses. Classes were divided among five yoga instructors. They participated in manual development, attended staff meetings throughout the study to maintain protocol fidelity, and taught the classes by the manual. The PI completed written fidelity assessments of each instructor's adherence to the protocol throughout the study.

### Statistics

Study data were analyzed using Stata/SE v13.1 (Stata Corp., College Station, TX).^14^ Unadjusted bivariate analyses used Fisher's exact test for categorical data and two-sample *t*-tests for continuous data. Generalized estimating equations (GEE) were used to analyze correlated longitudinal data from repeated measures on subjects across time.^17^ *p*-Values derived for all statistical comparisons and power analyses were two-tailed, using an alpha of 0.05 for statistical significance. Cohen's *d* was used to estimate effect sizes, and confidence intervals were calculated.

## Results

### Recruitment and demographics

**FIG. 1.**
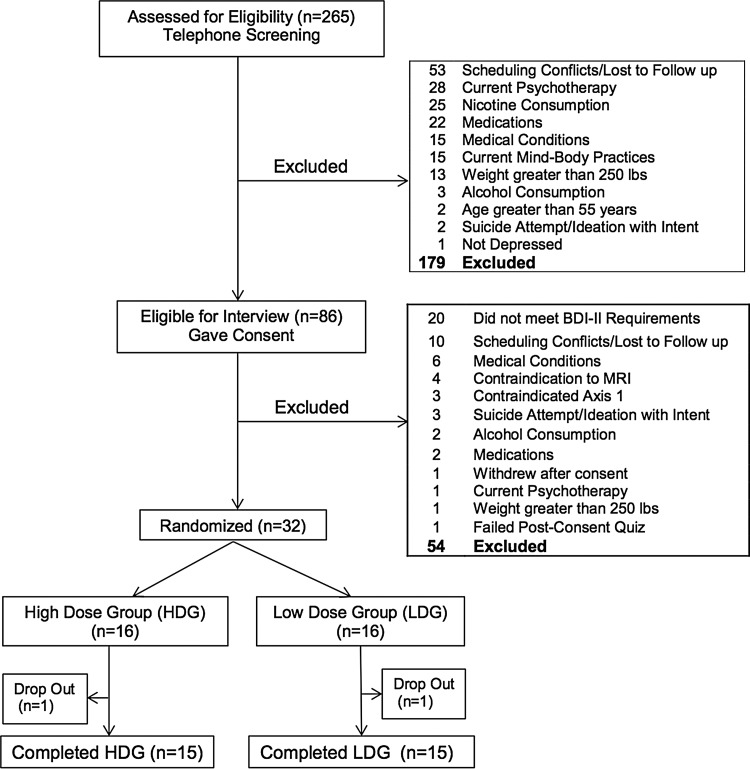
Flowchart of subject recruitment through completion.

Telephone screening yielded 265 subjects of whom 86 participated in a screening visit, 32 were randomized, with 30 completers (Fig. 1). Completers participated in the screening and evaluations at weeks 4, 8, and 12, except one HDG subject who did not complete the 8-week evaluation. In addition to MDD, subjects met criteria for the following Axis I anxiety disorders: eight post-traumatic stress disorder (PTSD) current, one PTSD in full remission, four PTSD subthreshold, three panic disorder in partial remission, one panic disorder in full remission, and one current social phobia. No subjects were excluded for anxiety disorders.

There were no significant differences between the HDG (*n* = 15; *M*_age_ = 38.4 ± 15.1 years) and LDG (*n* = 15; *M*_age_ = 34.7 ± 10.4 years) on demographic variables (Table 1). Fisher's exact test between HDG and LDG was statistically non-significant for marital status (*p* = 0.84), employment (*p* = 0.66), and race (*p* = 0.30).

**Table 1.** Demographics: HDG Versus LDG

*Demographic*	*HDG (*n* = 15)*	*LDG (*n* = 15)*	p
Age (years)	38.4 ± 15.1	34.7 ± 10.4	0.44
Female	13/15 (87%)	12/15 (80%)	1.00
Has children	5/15 (33%)	5/15 (33%)	1.00
Education (years)	16.3 ± 2.2	16.7 ± 2.2	0.62
*Marital status*			0.84
Married	2/15 (13%)	1/15 (7%)	
Cohabitating	2/15 (13%)	2/15 (13%)	
Divorced	3/15 (20%)	1/15 (7%)	
Separated	1/15 (7%)	1/15 (7%)	
Never married	7/15 (47%)	10/15 (67%)	
*Employment status*			0.66
Full-time	7/15 (47%)	5/15 (33%)	
Part-time regular hours	1/15 (7%)	2/15 (13%)	
Part-time irregular hours	3/15 (20%)	1/15 (7%)	
Student	3/15 (20%)	3/15 (20%)	
Unemployed	1/15 (7%)	3/15 (20%)	
*Race*			0.30
Caucasian	10/15 (67%)	13/15 (87%)	
Hispanic	0/15 (0%)	0/15 (0%)	
Asian	3/15 (20%)	0/15 (0%)	
Black or African American	2/15 (13%)	2/15 (13%)	

HDG, high-dose group; LDG, low-dose group.

### Yoga dose, compliance, and depressive symptoms

The assigned classes and homework (prescribed dose), the performed postures or breathing (time in minutes), and the compliance (performed/assigned) for yoga classes, postures homework, breathing homework, total homework, and total yoga time for HDG and LDG are listed in Table 2. The HDG had a significantly higher dose of yoga, measured by number of classes (*p* = 0.001), total homework (*p* = 0.05), and total minutes (*p* = 0.001). Two-sample *t*-tests comparing compliance in classes, homework, and total yoga minutes across groups were not statistically significant, consistent with both groups being comparably compliant with the protocol. Fidelity assessments documented that yoga instructors were compliant with the protocol.

**Table 2.** Between-Group Comparison of Minutes Assigned to Yoga Classes and Homework, Minutes Performed, and Compliance

	*Group*		
*Practice*	*HDG*	*LDG*	t*-test* p*-value*
*Classes (minutes assigned)*	3240	2160	
Attendance^*^	2532 ± 652	1818 ± 432	0.001
Compliance^**^	78 ± 20%	84 ± 20%	0.42
*Homework (minutes assigned)*	1440	1080	
Performed^*^	1543 ± 1095	919 ± 475	0.05
Compliance^**^	107 ± 76%	85 ± 44%	0.34
*Postures (minutes assigned)*	720	540	
Performed^*^	997 ± 910	556 ± 294	0.08
Compliance^**^	138 ± 126%	103 ± 54%	0.33
*Breathing (minutes assigned)*	720	540	
Performed^*^	546 ± 349	363 ± 230	0.10
Compliance^**^	76 ± 48%	67 ± 43%	0.61
*Total minutes (assigned)*	4680	3240	
Total minutes performed^*^	4075 ± 1314	2737 ± 625	0.001
Overall compliance^**^	87 ± 28%	84 ± 19%	0.77

^*^ Reported in minutes; mean ± standard deviation.

^**^ Reported as percentage.

**FIG. 2.**
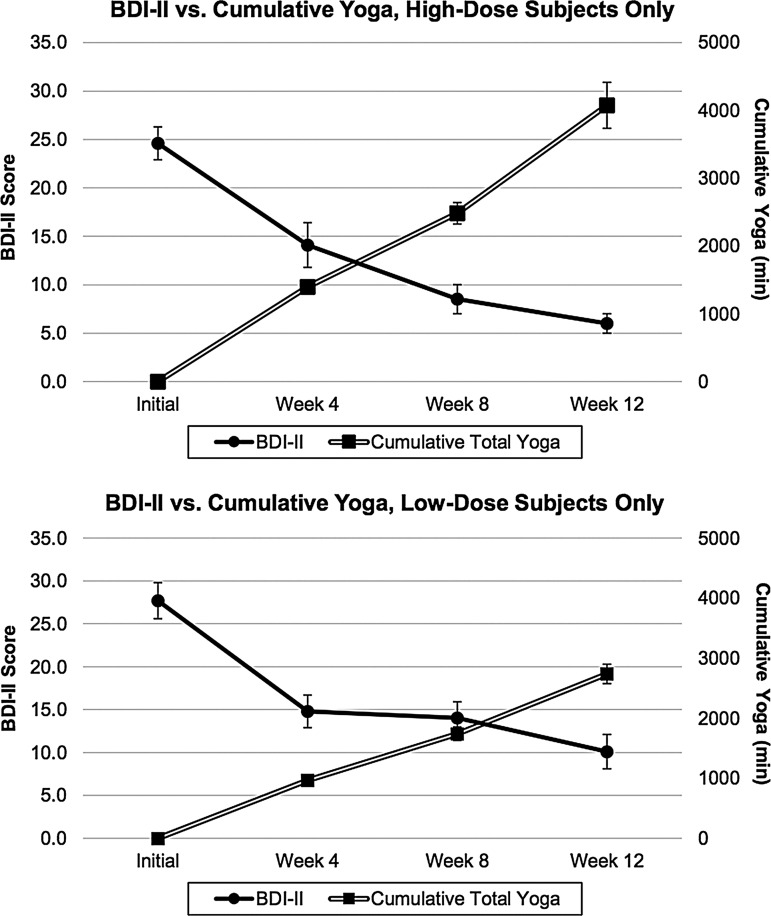
Beck Depression Inventory-II (BDI-II) versus Cumulative Yoga Minutes for high-dose group (HDG) and low-dose group (LDG) (mean ± SEM).

As shown in Table 3 and Figure 2, BDI-II scores declined significantly from screening to week 12 in the HDG (–18.6 ± 6.6; *t* = –10.9; *df* = 14; *p* < 0.001; Cohen's *d* = –2.81; 95% confidence interval [CI] −22.3 to −14.9) and from screening to week 12 in the LDG (–17.7 ± 9.3; *t* = –7.3; *df* = 14; *p* < 0.001; Cohen's *d =* –1.89; 95% CI –22.8 to –12.5). A two-sample *t*-test comparing BDI-II scores from HDG and LDG at screening was not statistically significant (*t* = –1.16; *df* = 28; *p* = 0.26), indicating no difference in the severity of depression between groups prior to randomization. BDI-II scores by group and time are provided in Table 3.

**Table 3.** Means and Standard Deviations for BDI-II Total Scores and Cumulative Yoga Minutes for HDG and LDG

*HDG*			*LDG*	
*Event*	*BDI-II total score*	*Cumulative total yoga minutes*	*BDI-II total score*	*Cumulative total yoga minutes*
Screening	24.6 ± 6.7	0	27.7 ± 8.0	0
Week 4	14.1 ± 8.9	1398 ± 294	14.8 ± 7.4	968 ± 225
Week 8	8.5 ± 5.7	2483 ± 611	14.0 ± 7.4	1741 ± 434
Week 12	6.0 ± 3.8	4075 ± 1314	10.1 ± 7.9	2737 ± 625

BDI-II, Beck Depression Inventory-II.

Changes in BDI-II scores and cumulative yoga minutes by group during the intervention are illustrated in Figure 2. A GEE regression model with BDI-II scores as the dependent variable and cumulative yoga minutes as the independent variable demonstrates negative regression coefficients, consistent with increasing total yoga minutes being inversely correlated with decreasing BDI-II scores in the LDG (β = –0.006; *z* = –3.68; *p* < 0.001) and the HDG (β = –0.001; *z* = –1.01; *p* = 0.31). A *t*-test comparing the BDI-II change scores between groups was not statistically significant (*t* = –0.32; *df* = 28; *p* = 0.75). The combination of significantly greater total yoga minutes in the HDG with no increased difference in the change in BDI-II score makes the estimate regression coefficient smaller, consistent with a lack of statistical significance for the GEE in the HDG.

A two-sided Fisher's exact test comparison showed the following statistically non-significant differences between groups from screening to week 12 in the proportion of responders (>50% decrease in BDI-II; 87% of HDG [13/15] and 73% of LDG [11/15]; *p* = 0.65), and remitters (BDI-II scores <14 at week 12; 93% of HDG [14/15] and 87% of LDG [13/15]; *p* = 1.00). Statistically significant differences in BDI-II scores ≤10 were found at week 12: 93% of HDG (14/15) and 53% of LDG (8/15; *p* = 0.04). Two subjects who were on a stable dose of antidepressants (venlafaxine and bupropion) had BDI-II scores that decreased from screening (38 and 16) to week 12 (14 and 9), respectively.

### Adverse events

Adverse events were assessed weekly using subject-completed adverse event forms and by a physician or clinical psychologist using an adverse event form at weeks 4, 8, and 12. No serious adverse events were associated with the yoga protocol. The most common adverse event clearly related to the intervention, reported in 13 subjects, was transient muscle soreness. Weekly adverse event sheets, subject reports of difficulties to staff, or staff observation triggered clinical evaluations. No subjects developed suicidal ideation during the study, and no subjects were withdrawn from the study. Two randomized subjects, one from each group, stopped attending classes and were lost to follow up prior to the 4-week evaluation. By the end of the study, two subjects had chosen to start psychotherapy, and none started antidepressants. Two subjects continued to meet the criteria for severe and moderately severe depression at end of the study, based on BDI-II scores at screening of 40 and 22 and at week 12 of 33 and 23, respectively. Although they still met the criteria for depression, both subjects stated that the intervention had been helpful and that they intended to continue classes. Because one subject in the HDG reported distressing thoughts while practicing coherent breathing alone at home, homework was changed for this individual to 30 min of postures with no coherent breathing, which reportedly alleviated the distress.

## Discussion

Although the HDG received a statistically significantly greater dose of yoga minutes (measured in yoga classes, homework, and total yoga minutes) compared with the LDG, there was no significant difference in response (>50% reduction in BDI-II scores) or remission (BDI-II scores <14) rates between the groups from screening to week 12. However, more HDG subjects had BDI-II scores <10 at week 12, demonstrating less depressive symptomatology. Results indicate that both groups completed sufficient intervention minutes to improve rates of remission and response significantly, but that the higher dose may be more effective in reducing BDI-II scores to <10.

These findings are consistent with a previous yoga study by the authors' group,^18^ in which subjects with no history of psychiatric illness completed an average of two classes and one homework session per week, which was associated with significant improvements in mood and decreased anxiety in yoga subjects in both within group analyses and in comparison to a walking control group. The dose in the previously published study is similar to the dose of the current LDG.

Drug dosing is based on drug half-life, the time required for a substance to decrease by half. Similarly, it is possible that the yoga intervention in the present study has a half-life of 48 h, such that an intervention dosed at three times a week (e.g., two classes plus one homework session) would be sufficient to improve mood symptoms. Although the subjects in both groups were compliant, those in the HDG reported that three weekly classes entailed a demanding time commitment, as did subjects who declined to participate in the study due to the time commitment. Considering the similar response and remission rates, the lighter schedule of the LDG may better balance effective intervention frequency with time demands.

The Yoga Sutras of Patanjali described eight limbs of yoga.^19^ Treatment trials often include limb 3 (postures/asanas), limb 4 (breathing exercises/pranayama), limb 6 (concentration/dharana), and limb 7 (meditation/dhyana). According to Patanjali, postures, breathing, and concentration precede meditation as a way to prepare the body and mind to sit without distraction. Given that symptoms of depression include impaired concentration, this study provided subjects with concrete activities on which to focus in the form of postures and breathing exercises, thereby including components of concentration.

The quality of the intervention was strengthened by (1) use of a treatment manual, (2) rigorous training and certification for yoga instructors, including ongoing study with the same teacher during this trial, (3) participation of instructors in developing the yoga treatment manual, (4) training in coherent breathing and use of a CD for pacing, and (5) fidelity assessments of yoga instructors' adherence to the protocol.

The results of this study must be interpreted with caution in the context several limitations, for example the small sample size and lack of an active non-yoga control (both groups received Iyengar yoga plus coherent breathing). Also, the supportive group environment and multiple subject interactions with research staff each week could have contributed to the reduction in depressive symptoms. Accordingly, a larger RCT with a walking comparison group is underway. This study enrolled subjects with MDD who had a low risk of self-injury under the supervision of trained clinicians. Thus, the results cannot be generalized to MDD with more acute suicidality or more severe symptoms. This intervention was not designed to be a substitute for the evaluation and treatment of depression by trained professionals.

No subjects who were on antidepressant medication changed their medication during this study. The two subjects on antidepressant medication were depressed at screening and improved over the course of the intervention. This provides preliminary support for the use of yoga-based interventions as an adjunct to pharmacologic treatment for depression. Compared with psychotropic augmentation of antidepressant medication, this intervention has the advantages of avoiding additional drug side effects and drug–drug interactions. A larger group of patients on antidepressant drugs would be needed to validate this observed augmentation effect.

## Conclusions

This dosing study provides evidence that participation in an intervention composed of Iyengar yoga and coherent breathing is associated with a significant reduction in depressive symptoms for individuals with MDD, both on and off antidepressant medications. The HDG and LDG showed no significant differences in compliance or in rates of response or remission. Although the HDG had significantly more subjects with BDI-II scores ≤10 at week 12, twice weekly classes (plus home practice) may rates of response or remission. Although the HDG, thrice weekly classes (plus home practice) had significantly more subjects with BDI-II scores ≤10 at week 12, the LDG, twice weekly classes (plus home practice) may constitute a less burdensome but still effective way to gain the mood benefits from the intervention. This study supports the use of an Iyengar yoga and coherent breathing intervention as a treatment to alleviate depressive symptoms in MDD.

## Supplementary Material

Supplemental data

Supplemental data

Supplemental data
