# Supplementary material for: Treatment of Major Depressive Disorder with Iyengar Yoga and Coherent Breathing: A Randomized Controlled Dosing Study
Source: J Altern Complement Med. 2017 Mar 1;23(3):201–7. doi: 10.1089/acm.2016.0140 (PMC5359682; doi:10.1089/acm.2016.0140)
Supplement: Supplemental data [file Supp_Table1.pdf]

Supplementary Data

Supplementary Table 1. Detailed Description of 11 Unique Yoga Asana Classes Held Each Week of the 12-Week Intervention

Each class was 90 min, with approximately 60 min of Iyengar yoga postures, a transition from the posture section to the breathing section of 10 min that included deep relaxation (savasana) and instruction in Ujjayi breathing, followed by a 20-min coherent breathing exercise with equal inhalation and exhalation at five breaths per minute guided by an audio recording with a high tone for inhalation and a low tone for exhalation. Postures that are italicized and underlined are done if time allows. The Sutra translation and comment at the beginning of each unique intervention is excerpted from *Light on the Yoga Sutras of Patanjali* (Iyengar, 1993).

Week 1:
Sutra II.29
Seated/Reclining/Breathing Instruction: (1) Cross-Legged Easy Pose with Upward Hand Pose ×2 and Upward Bound Knuckle Pose ×2 (alternating top index finger); (2) *Supine Hand to Big Toe Pose I*; (3) *Supine Hand to Big Toe Pose II*; (4) Seated Forward Bend to Plow Pose Cycle: Starting in Staff Pose inhale, then exhale to Plow Pose, inhale to Staff Pose, and then exhale to Seated Forward Bend.
Sun Salutation (Practice each pose separately, then combine. Step between poses. The sequence provided below is used throughout the intervention. (1) Mountain Pose; (2) Upward Hand Pose (inhale); (3) Standing Forward Bend Pose—hands to floor or shins (exhale); (4) Standing Forward Bend Pose—head up (inhale); (5) Standing Forward Bend Pose—hands to floor or shins (exhale); (6) Bend knees in preparation (inhale); (7) Step or back to Downward Facing Dog Pose (exhale); (8) Downward Facing Dog Pose—take three breaths in this position; (9) bring shoulders over wrists (inhale; modification bend knees to the floor); (10) Four Limb Staff Pose (exhale; modification knees on floor); (11) Upward Facing Dog Pose (inhale; modification—Cobra Pose with bent elbows); (12) Downward Facing Dog Pose (exhale); (13) Downward Facing Dog Pose—take three breaths in this position; (14) Step to Standing Forward Bend Pose (inhale); (15) Standing Forward Bend Pose (exhale); (16) Standing Forward Bend Pose—head up (inhale); (17) Standing Forward Bend—hands to floor or shins (exhale); (18) Upward Hand Pose (inhale); (19) Mountain Pose with hands to heart (exhale).
Standing Poses: (1) Mountain Pose; (2) Chair Pose; (3) Tree Pose; (4) *Triangle Pose*; (5) *Warrior II*.
Back Bends: (1) Supported Supine Back Bend (use two blocks; one supporting thoracic spine, one supporting head); (2) Supported Bridge Pose (with bolster and block against the wall).
Transition Poses: (1) Supine Twist—Bent Knees Variation; (2) *Bharadvaja's Twist I*.
Forward Bends: Child's Pose.
Inversion: Inverted Pose.
Deep Relaxation/Breathing Instruction: Corpse Pose.

Week 2:
Sutra II.30
Seated/Reclining Poses/Breathing Instruction: (1) Cross-Legged Easy Pose; (2) Bound Angle Pose.
Sun Salutation (Step between poses. Use posture sequence 1–18 in Week 1).
Standing Poses: (1) Mountain Pose with Upward Hand Pose ×2 and Upward Bound Knuckle Pose ×2; (2) Warrior II; (3) *Side Angle Pose*; (4) *Wide Legged Standing Forward Bend Pose*.
Back Bends: (1) Supine Bound Angle Pose (Back Bend with blankets in cross, two folded and stacked blankets under the spine and one across for the head); (2) Bridge Pose—Bent Leg Variation; (3) Supported Bridge Pose (with bolster and block against the wall).
Transition Poses: (1) Supine Twist—Bent Knees Variation; (2) *Marichi's Twist I*.
Forward Bends: (1) *Head to Knee Pose*, (2) Seated Forward Bend Pose.
Inversion: Inverted Pose.
Deep Relaxation/Breathing Instruction: Corpse Pose.

Week 3:
Sutra II.32
Seated/Reclining Poses/Breathing Instruction: (1) Cross-Legged Easy Pose with Upward Hand Pose ×2 and Upward Bound Knuckle Pose ×2; (2) Bound Angle Pose; (3) *Hero Pose (Sit on block if needed, knees facing forward)*; (4) Seated Forward Bend to Plow Pose Cycle (use breath instruction in Week 1).
Sun Salutation (Step between poses. Use posture sequence 1–18 in Week 1).
Standing Poses: (1) Mountain Pose; (2) *Tree Pose*; (3) Triangle Pose; (4) Half Moon Pose; (5) *Wide Legged Standing Forward Bend Pose*.
Back Bends: (1) Supine Bound Angle Pose (Back Bend with blankets in cross, two folded and stacked blankets under the spine and one across for the head); (2) Supported Supine Back Bend; (3) Bridge Pose—Bent Leg Variation.
Transition Pose/Twist: (1) Supine Hug Knees to Chest; (2) *Hero Pose with Twist (Sit on block if needed).*
Forward Bends: Child's Pose.
Inversion: Inverted Pose.
Deep Relaxation/Breathing Instruction: Corpse Pose.

(*continued*)

Week 4:
Sutra II.46
Seated/Reclining Poses/Breathing Instruction: (1) Hero Pose (Sit on block if needed) with Upward Bound Knuckle, Pose ×2 and *Cow Face Pose (Arms)*; (2) Supine Hand to Big Toe Pose I (Leg vertically up); (3) Supine Hand to Big Toe Pose II (Leg out to the side).
Sun Salutation (Step between poses. Use posture sequence 1–18 in Week 1).
Standing Poses: (1) Mountain Pose; (2) *Triangle Pose*; (3) Warrior I; (4) Intense Side
Stretch Pose with Reverse Hand Salute—(Practice with hands to elbows first, advance those who can to Reverse Hand Salute).
Back Bends: (1) Camel Pose; (2) Supported Bridge Pose (with block under sacrum).
Transition Poses/Twist: Half Lord of the Fishes Pose I.
Forward Bends: (1) Child's Pose; (2) *Head to Knee Pose.*
Inversions: (1) Inverted Pose or (2) Plow Pose to Shoulderstand (Modification: Stack three blankets folded in half lengthwise and then in quarters with the folded edges aligned on edge of mat. Measure a leg distance from the wall by sitting on the edge of the blankets facing the wall, and extending legs to reach the wall. The subjects were instructed to lie on their back with shoulders on the folded edges of the blankets and head on the floor. While exhaling, the subjects brought their legs over head and such that the feet touched the wall or a chair. A block was placed under the sacrum to assist in lifting the hips off floor; Practice one-footed Shoulderstand with other foot on the wall first, advance those who can into full Shoulderstand).
At the end of week 4, students either continued to do Inverted Pose or were advanced as appropriate to Plow and Shoulderstand poses. Plow and Shoulderstand were modified as above to avoid stress on the neck.
Deep Relaxation/Breathing Instruction: Corpse Pose.

Week 5:
Sutra II.47
Seated/Reclining Poses/Breathing Instruction: (1) Cross-Legged Easy Pose with Upward Hand Pose ×2 and Upward Bound Knuckle Pose ×2; (2) *Supine Hand to Big Toe Pose I*; (3) *Upward Extended Legs Pose.*
Sun Salutation (Step between poses. Use posture sequence 1–18 in Week 1).
Standing Poses: (1) Mountain Pose; (2) *Warrior II*; (3) Triangle Pose; (4) Revolved Triangle Pose (facing forward, block between the feet, step left foot back, raise left arm and bend forward, place the left hand on the block).
Back Bends: (1) Supported Supine Back Bend (two blocks); (2) Upward Facing Dog Pose; (3) Camel Pose.
Transition Poses/Twist: (1) Child's Pose; (2) Marichi's Twist I.
Forward Bends: Seated Wide Angle Pose.
Inversions: Plow Pose to Shoulderstand (use modification in week 4), or Inverted Pose.
Deep Relaxation/Breathing Instruction: Corpse Pose.

Week 6:
Sutra II.49
Seated/Reclining Poses/Breathing Instruction: (1) Hero Pose with Upward Hand Pose ×2 and Cow Face Pose (Arms); (2) Bound Angle Pose; (3) Seated Forward Bend to Plow Pose Cycle (use breath instruction in Week 1).
Sun Salutation: Teach jumping technique. (Step or jump between poses. Use posture sequence 1–18 in Week 1).
Standing Poses: (1) Mountain Pose with Upward Hand Pose and *Reverse Hand Salute*; (2) *Chair Pose*; (3) Warrior I; (4) Intense Side Stretch Pose.
Back Bends: (1) Supine Bound Angle Pose (Back Bend with blankets in cross, two folded and stacked blankets under the spine and one across for the head); (2) Bridge Pose—Bent Leg Variation; (3) Supported Bridge Pose (with bolster and block against the wall).
Transition Poses/Twists: Bharadvaja's Twist I.
Forward Bends: (1) Child's Pose; (2) *Seated Forward Bend Pose.*
Inversions: Plow Pose to Shoulderstand (use modification in week 4), or Inverted Pose.
Deep Relaxation/Breathing Instruction: Corpse Pose.

Week 7:
Sutra II.54
Seated/Reclining Poses/Breathing Instruction: (1) Cross-Legged Easy Pose with Upward Hand Pose ×2; (2) *Staff Pose*; (3) Bound Angle Pose.
Sun Salutation: Teach jumping technique. (Step or jump between poses. Use posture sequence 1–18 in Week 1).
Standing Poses: (1) Mountain Pose; (2) *Extended Hand to Big Toe Pose I*; (3) *Extended Hand to Big Toe Pose II*; (4) Intense Side Stretch Pose; (5) Revolved Triangle Pose (use instruction in Week 5).
Back Bends: (1) Standing Back Bend ×3, reach the arms up and back to the wall behind, hold the last one; (2) Supported Supine Back Bend with two blocks; (3) Bridge Pose—Bent Leg Variation; (4) Supported Bridge Pose (with block under sacrum).
Transition Poses/Twists: Bharadvaja's Twist I or *Marichi's Twist III.*
Forward Bends: (1) Child's Pose; (2) *Head to Knee Pose.*
Inversions: Plow Pose to Shoulderstand (use modification in week 4), or Inverted Pose.
Deep Relaxation/Breathing Instruction: Corpse Pose.

(*continued*)

Week 8:
Sutra III.1
Seated/Reclining Poses/Breathing Instruction: (1) Hero Pose; (2) Staff Pose; (3) *Full Boat Pose*; (4) *Half Boat Pose*
Sun Salutation (Step or jump between poses. Use posture sequence 1–18 in Week 1).
Standing Poses: (1) Mountain Pose; (2) *Half Moon Pose*; (3) Warrior I; (4) Warrior III.
Back Bends: (1) *Upward Facing Dog Pose*; (2) Camel Pose; (3) Supported Bridge Pose (with bolster and block against the wall).
Transition Poses/Twists: Marichi's Twist I.
Forward Bends: Child's Pose.
Inversions: Plow Pose to Shoulderstand (use modification in week 4), or Inverted Pose.
Deep Relaxation/Breathing Instruction: Corpse Pose.

Week 9:
Sutra III.2
Seated/Reclining Poses/Breathing Instruction: (1) Hero Pose with Upward Hand Pose ×2, Upward Bound Knuckle Pose ×2, and *Cow Face Pose (Arms)*; (2) Bound Angle Pose; (3) Seated Forward Bend to Plow Pose Cycle (use breath instruction in Week 1).
Sun Salutation (Step or jump between poses. Use posture sequence 1–18 in Week 1).
Standing Poses: (1) Mountain Pose; (2) Triangle Pose; (3) Side Angle Pose.
Back Bends: (1) Standing Back Bend; (2) Supine Bound Angle Pose (Back Bend with blankets in cross, two folded and stacked blankets under the spine and one across for the head); (3) Bridge Pose—Bent Leg Variation; (4) Supported Bridge Pose (with block under sacrum).
Transition Poses/Twists: Half Lord of the Fishes Pose I.
Forward Bend Poses: (1) Seated Wide Angle Pose; (2) *Standing Forward Bend Pose*.
Inversions: Plow Pose to Shoulderstand (use modification in week 4), or Inverted Pose.
Deep Relaxation/Breathing Instruction: Corpse Pose.

Week 10:
Sutra III.3
Seated/Reclining Poses/Breathing Instruction: (1) Cross-Legged Easy Pose; (2) Bound Angle Pose; (3) *Supine Hand to Big Toe Pose I*.
Sun Salutation (Step or jump between poses. Use posture sequence 1–18 in Week 1).
Standing Poses: (1) Mountain Pose; (2) Intense Side Stretch Pose; (3) Revolved Triangle Pose.
Back Bends: (1) Supine Bound Angle Pose (Back Bend with blankets in cross, two folded and stacked blankets under the spine and one across for the Head); (2) Supported Bridge Pose (with bolster and block against the wall).
Transition Poses/Twists: Marichi's Twist III.
Forward Bend Poses: (1) Child's Pose; (2) *Head to Knee Pose*.
Inversions: Plow Pose to Shoulderstand (use modification in week 4), or Inverted Pose.
Deep Relaxation/Breathing Instruction: Corpse Pose.

Weeks 11 and 12:
Sutra I.2
Seated/Reclining Poses/Breathing Instruction: (1) Cross-Legged Easy Pose with Upward Hand Pose ×3 and Upward Bound Knuckle Pose ×2. (2) Seated Forward Bend to Plow Pose Cycle, ×8 with bent knees, ×8 with straight legs (use breath instruction in Week 1).
Sun Salutation ×3: (Step or jump between poses. Use posture sequence 1–18 in Week 1).
Standing Poses: (1) Tree Pose ×2 each side; (2) Warrior II Pose ×2 each side; (3) Triangle Pose ×2 each side; (4) Half Moon Pose ×2 each side; (5) Revolved Triangle Pose ×2 each side.
Back Bends: (1) Standing Backbend ×3; (2) Supported Supine Back Bend; (3) Bridge Pose Bent Leg Variation ×4; (4) Camel Pose ×3; (5) Child's Pose; (6) Supported Bridge Pose (with bolster and block against the wall).
Transition Poses: Supine Twist—Bent Knees Variation ×2 each side.
Forward Bends: (1) Head to Knee Pose; (2) Seated Forward Bend Pose.
Inversions: Plow Pose to Shoulderstand (use modification in week 4), or Inverted Pose.
Transition Poses/Twists: Supine Twist—Bent Knees Variation ×2 each side.
Deep Relaxation/Breathing Instruction: Corpse Pose.
