# Supplementary material for: Treatment of Major Depressive Disorder with Iyengar Yoga and Coherent Breathing: A Randomized Controlled Dosing Study
Source: J Altern Complement Med. 2017 Mar 1;23(3):201–7. doi: 10.1089/acm.2016.0140 (PMC5359682; doi:10.1089/acm.2016.0140)
Supplement: Supplemental data [file Supp_Table2.pdf]

Supplementary Table 2. English and Sanskrit Names of Postures Listed By Intervention Week

All Sanskrit names and posture descriptions are taken from Iyengar BKS: *Yoga The Path to Holistic Health* (Dorling Kindersley, 2008), except those listed below with an asterisk (*) following the Sanskrit name, which are taken from Mehta S, Mehta M, Mehta S: *Yoga The Iyengar Way* (Dorling Kindersley, 1990). The commonly used English names of the following poses are used: Adho Mukha Virasana or Virasana Forward Bend is referred to as Child's Pose, Prasarita Padottanasana or Intense Leg Stretch is referred to as Wide Legged Standing Forward Bend, Paschimottanasana or Intense Back Stretch is referred to as Seated Forward Bend, Tadasana Paschima Namaskarasana, or Mountain Pose with Hands Folded Behind the Back is referred to as Reverse Hand Salute, Tadasana Urdhva Baddhanguliasana, or Mountain Pose with Fingers Interlocked is referred to as Upward Bound Knuckle Pose—Standing, Utthita Parsvakonasana or Extended Side Angle Pose is referred to as Side Angle Pose, and Utthita Trikonasana or Extended Triangle Pose is referred to as Triangle Pose.

English name of poses	*Sanskrit name of Asanas*	*Weeks*										
		1	*2*	*3*	*4*	*5*	*6*	*7*	*8*	*9*	*10*	*11*
Seated/Reclining Poses												
Bound Angle	Baddha Konasana*		x	x			x	x		x	x	
Cow Face (Arms)	Gomukhasana (Arms)*				x		x			x		
Full Boat	Paripurna Navasana								x			
Half Boat	Ardha Navasana*								x			
Hero	Virasana			x	x		x		x	x		
Cross-Legged Easy	Sukhasana*	x	x	x		x		x			x	x
Seated Forward Bend to Plow Cycle	Paschimottanasana/Halasana Vinyasa	x		x			x			x		x
Staff	Dandasana							x	x			
Supine Bound Angle	Supta Baddha Konasana			x								
Supine Hand to Big Toe I	Supta Padangusthasana I*	x			x	x					x	
Supine Hand to Big Toe II	Supta Padangusthasana II*	x			x							
Upward Bound Knuckle -Seated	Parvatasana*	x		x		x				x		x
Upward Extended Legs	Urdhva Prasarita Padasana*					x						
Upward Hand - Seated	Urdhva Hastasana Variation*	x		x		x	x	x		x		x
Sun Salutation Variation	Surya Namaskar Variation*											
Mountain	Tadasana	x	x	x	x	x	x	x	x	x	x	x
Upward Hand	Urdhva Hastasana*	x	x	x	x	x	x	x	x	x	x	x
Standing Forward Bend	Uttanasana	x	x	x	x	x	x	x	x	x	x	x
Standing Forward Bend - Head Up	Uttanasana - Head Up	x	x	x	x	x	x	x	x	x	x	x
Downward Facing Dog	Adho Mukha Svanasana	x	x	x	x	x	x	x	x	x	x	x
Four Limb Staff	Caturanga Dandasana*	x	x	x	x	x	x	x	x	x	x	x
Upward Facing Dog	Urdhva Mukha Svanasana*	x	x	x	x	x	x	x	x	x	x	x
Downward Facing Dog	Adho Mukha Svanasana	x	x	x	x	x	x	x	x	x	x	x
Standing Forward Bend- Head Up	Uttanasana - Head Up	x	x	x	x	x	x	x	x	x	x	x
Standing Forward Bend	Uttanasana	x	x	x	x	x	x	x	x	x	x	x
Upward Hand	Urdhva Hastasana*	x	x	x	x	x	x	x	x	x	x	x
Mountain	Tadasana											
Standing Poses												
Chair	Utkatasana*	x					x					
Extended Hand to Big Toe I	Utthita Hasta Padangusthasana I*							x				
Extended Hand to Big Toe II	Utthita Hasta Padangusthasana II*							x				
Wide Legged Standing Forward Bend	Prasarita Padottanasana		x	x								
Side Angle	Utthita Parsvakonasana		x							x		
Half Moon	Ardha Chandrasana			x					x			x
Intense Side Stretch	Parsvottanasana				x		x	x			x	
Mountain	Tadasana	x	x	x	x	x	x	x	x	x	x	x
Reverse Hand Salute	Tadasana Paschima Namaskarasana				x		x					
Revolved Triangle	Parivrtta Trikonasana*					x		x			x	x
Tree	Vrksasana*	x		x								x
Triangle	Utthita Trikonasana	x		x	x	x			x	x		x
Upward Bound Knuckle - Standing	Tadasana Urdhva Baddhanguliasana		x									
Upward Hand	Urdhva Hastasana*		x				x					
Warrior I	Virabhadrasana I				x		x		x			
Warrior II	Virabhadrasana II	x	x			x						x
Warrior III	Virabhadrasana III*								x			

(*continued*)

Supplementary Table 2. (Continued)

English name of poses	*Sanskrit name of Asanas*	*Weeks*										
		1	*2*	*3*	*4*	*5*	*6*	*7*	*8*	*9*	*10*	*11*
Back Bends												
Setu Bandha Sarvangasana Variation		x	x			x	x		x		x	
Camel	Ustrasana				x	x			x			x
Cobra	Bhujangasana*											x
Standing Back Bend	Urdhva Dhanurasana Prep at Wall*							x		x		x
Supine Bound Angle—Back Bend	Supta Baddha Konasana		x	x			x			x	x	
Supported Bridge—with bolster	Setu Bandha Sarvangasana Variation	x	x				x		x		x	x
Supported Bridge—with block	Setu Bandha Sarvangasana Variation				x			x		x		
Supported Supine Back Bend—with two blocks	Matsyasana Variation*	x		x		x		x				x
Upward Facing Dog	Urdhva Mukha Svanasana*					x			x			
Twists												
Half Lord of the Fishes I	Ardha Matsyendrasana I*				x					x		
Marichi's Twist I	Maricyasana I*		x			x			x			
Marichi's Twist III	Maricyasana III*							x			x	
Bharadvaja's Twist I	Bharadvajasana I*	x					x	x				
Supine Twist—Bent Knees Variation	Jathara Parivartanasana Variation*	x	x									x
Hero with Twist	Parsva Virasana			x								
Forward Bends												
Child's	Adho Mukha Virasana*	x		x	x	x	x	x	x		x	x
Head to Knee	Janu Sirsasana		x		x			x			x	x
Seated Forward Bend	Paschimottanasana		x				x					x
Seated Wide Angle	Upavista Konasana					x				x		
Inversions												
Inverted Pose	Viparita Karani	x	x	x	x	x	x	x	x	x	x	x
Plow	Halasana*				x	x	x	x	x	x	x	x
Shoulderstand	Salamba Sarvangasana*				x	x	x	x	x	x	x	x
Deep Relaxation												
Corpse	Savasana	x	x	x	x	x	x	x	x	x	x	x
