# Supplementary material for: Treatment of Major Depressive Disorder with Iyengar Yoga and Coherent Breathing: A Randomized Controlled Dosing Study
Source: J Altern Complement Med. 2017 Mar 1;23(3):201–7. doi: 10.1089/acm.2016.0140 (PMC5359682; doi:10.1089/acm.2016.0140)
Supplement: Supplemental data [file Supp_Data.pdf]

Supplementary Data

Supplementary Table 1. Detailed Description of 11 Unique Yoga Asana Classes Held Each Week of the 12-Week Intervention

Each class was 90 min, with approximately 60 min of Iyengar yoga postures, a transition from the posture section to the breathing section of 10 min that included deep relaxation (savasana) and instruction in Ujjayi breathing, followed by a 20-min coherent breathing exercise with equal inhalation and exhalation at five breaths per minute guided by an audio recording with a high tone for inhalation and a low tone for exhalation. Postures that are italicized and underlined are done if time allows. The Sutra translation and comment at the beginning of each unique intervention is excerpted from *Light on the Yoga Sutras of Patanjali* (Iyengar, 1993).

Week 1:
Sutra II.29
Seated/Reclining/Breathing Instruction: (1) Cross-Legged Easy Pose with Upward Hand Pose ×2 and Upward Bound Knuckle Pose ×2 (alternating top index finger); (2) *Supine Hand to Big Toe Pose I*; (3) *Supine Hand to Big Toe Pose II*; (4) Seated Forward Bend to Plow Pose Cycle: Starting in Staff Pose inhale, then exhale to Plow Pose, inhale to Staff Pose, and then exhale to Seated Forward Bend.
Sun Salutation (Practice each pose separately, then combine. Step between poses. The sequence provided below is used throughout the intervention. (1) Mountain Pose; (2) Upward Hand Pose (inhale); (3) Standing Forward Bend Pose—hands to floor or shins (exhale); (4) Standing Forward Bend Pose—head up (inhale); (5) Standing Forward Bend Pose—hands to floor or shins (exhale); (6) Bend knees in preparation (inhale); (7) Step or back to Downward Facing Dog Pose (exhale); (8) Downward Facing Dog Pose—take three breaths in this position; (9) bring shoulders over wrists (inhale; modification bend knees to the floor); (10) Four Limb Staff Pose (exhale; modification knees on floor); (11) Upward Facing Dog Pose (inhale; modification—Cobra Pose with bent elbows); (12) Downward Facing Dog Pose (exhale); (13) Downward Facing Dog Pose—take three breaths in this position; (14) Step to Standing Forward Bend Pose (inhale); (15) Standing Forward Bend Pose (exhale); (16) Standing Forward Bend Pose—head up (inhale); (17) Standing Forward Bend—hands to floor or shins (exhale); (18) Upward Hand Pose (inhale); (19) Mountain Pose with hands to heart (exhale).
Standing Poses: (1) Mountain Pose; (2) Chair Pose; (3) Tree Pose; (4) *Triangle Pose*; (5) *Warrior II*.
Back Bends: (1) Supported Supine Back Bend (use two blocks; one supporting thoracic spine, one supporting head); (2) Supported Bridge Pose (with bolster and block against the wall).
Transition Poses: (1) Supine Twist—Bent Knees Variation; (2) *Bharadvaja's Twist I*.
Forward Bends: Child's Pose.
Inversion: Inverted Pose.
Deep Relaxation/Breathing Instruction: Corpse Pose.

Week 2:
Sutra II.30
Seated/Reclining Poses/Breathing Instruction: (1) Cross-Legged Easy Pose; (2) Bound Angle Pose.
Sun Salutation (Step between poses. Use posture sequence 1–18 in Week 1).
Standing Poses: (1) Mountain Pose with Upward Hand Pose ×2 and Upward Bound Knuckle Pose ×2; (2) Warrior II; (3) *Side Angle Pose*; (4) *Wide Legged Standing Forward Bend Pose*.
Back Bends: (1) Supine Bound Angle Pose (Back Bend with blankets in cross, two folded and stacked blankets under the spine and one across for the head); (2) Bridge Pose—Bent Leg Variation; (3) Supported Bridge Pose (with bolster and block against the wall).
Transition Poses: (1) Supine Twist—Bent Knees Variation; (2) *Marichi's Twist I*.
Forward Bends: (1) *Head to Knee Pose*, (2) Seated Forward Bend Pose.
Inversion: Inverted Pose.
Deep Relaxation/Breathing Instruction: Corpse Pose.

Week 3:
Sutra II.32
Seated/Reclining Poses/Breathing Instruction: (1) Cross-Legged Easy Pose with Upward Hand Pose ×2 and Upward Bound Knuckle Pose ×2; (2) Bound Angle Pose; (3) *Hero Pose (Sit on block if needed, knees facing forward)*; (4) Seated Forward Bend to Plow Pose Cycle (use breath instruction in Week 1).
Sun Salutation (Step between poses. Use posture sequence 1–18 in Week 1).
Standing Poses: (1) Mountain Pose; (2) *Tree Pose*; (3) Triangle Pose; (4) Half Moon Pose; (5) *Wide Legged Standing Forward Bend Pose*.
Back Bends: (1) Supine Bound Angle Pose (Back Bend with blankets in cross, two folded and stacked blankets under the spine and one across for the head); (2) Supported Supine Back Bend; (3) Bridge Pose—Bent Leg Variation.
Transition Pose/Twist: (1) Supine Hug Knees to Chest; (2) *Hero Pose with Twist (Sit on block if needed).*
Forward Bends: Child's Pose.
Inversion: Inverted Pose.
Deep Relaxation/Breathing Instruction: Corpse Pose.

(*continued*)

Week 4:
Sutra II.46
Seated/Reclining Poses/Breathing Instruction: (1) Hero Pose (Sit on block if needed) with Upward Bound Knuckle, Pose ×2 and *Cow Face Pose (Arms)*; (2) Supine Hand to Big Toe Pose I (Leg vertically up); (3) Supine Hand to Big Toe Pose II (Leg out to the side).
Sun Salutation (Step between poses. Use posture sequence 1–18 in Week 1).
Standing Poses: (1) Mountain Pose; (2) *Triangle Pose*; (3) Warrior I; (4) Intense Side
Stretch Pose with Reverse Hand Salute—(Practice with hands to elbows first, advance those who can to Reverse Hand Salute).
Back Bends: (1) Camel Pose; (2) Supported Bridge Pose (with block under sacrum).
Transition Poses/Twist: Half Lord of the Fishes Pose I.
Forward Bends: (1) Child's Pose; (2) *Head to Knee Pose.*
Inversions: (1) Inverted Pose or (2) Plow Pose to Shoulderstand (Modification: Stack three blankets folded in half lengthwise and then in quarters with the folded edges aligned on edge of mat. Measure a leg distance from the wall by sitting on the edge of the blankets facing the wall, and extending legs to reach the wall. The subjects were instructed to lie on their back with shoulders on the folded edges of the blankets and head on the floor. While exhaling, the subjects brought their legs over head and such that the feet touched the wall or a chair. A block was placed under the sacrum to assist in lifting the hips off floor; Practice one-footed Shoulderstand with other foot on the wall first, advance those who can into full Shoulderstand).
At the end of week 4, students either continued to do Inverted Pose or were advanced as appropriate to Plow and Shoulderstand poses. Plow and Shoulderstand were modified as above to avoid stress on the neck.
Deep Relaxation/Breathing Instruction: Corpse Pose.

Week 5:
Sutra II.47
Seated/Reclining Poses/Breathing Instruction: (1) Cross-Legged Easy Pose with Upward Hand Pose ×2 and Upward Bound Knuckle Pose ×2; (2) *Supine Hand to Big Toe Pose I*; (3) *Upward Extended Legs Pose.*
Sun Salutation (Step between poses. Use posture sequence 1–18 in Week 1).
Standing Poses: (1) Mountain Pose; (2) *Warrior II*; (3) Triangle Pose; (4) Revolved Triangle Pose (facing forward, block between the feet, step left foot back, raise left arm and bend forward, place the left hand on the block).
Back Bends: (1) Supported Supine Back Bend (two blocks); (2) Upward Facing Dog Pose; (3) Camel Pose.
Transition Poses/Twist: (1) Child's Pose; (2) Marichi's Twist I.
Forward Bends: Seated Wide Angle Pose.
Inversions: Plow Pose to Shoulderstand (use modification in week 4), or Inverted Pose.
Deep Relaxation/Breathing Instruction: Corpse Pose.

Week 6:
Sutra II.49
Seated/Reclining Poses/Breathing Instruction: (1) Hero Pose with Upward Hand Pose ×2 and Cow Face Pose (Arms); (2) Bound Angle Pose; (3) Seated Forward Bend to Plow Pose Cycle (use breath instruction in Week 1).
Sun Salutation: Teach jumping technique. (Step or jump between poses. Use posture sequence 1–18 in Week 1).
Standing Poses: (1) Mountain Pose with Upward Hand Pose and *Reverse Hand Salute*; (2) *Chair Pose*; (3) Warrior I; (4) Intense Side Stretch Pose.
Back Bends: (1) Supine Bound Angle Pose (Back Bend with blankets in cross, two folded and stacked blankets under the spine and one across for the head); (2) Bridge Pose—Bent Leg Variation; (3) Supported Bridge Pose (with bolster and block against the wall).
Transition Poses/Twists: Bharadvaja's Twist I.
Forward Bends: (1) Child's Pose; (2) *Seated Forward Bend Pose.*
Inversions: Plow Pose to Shoulderstand (use modification in week 4), or Inverted Pose.
Deep Relaxation/Breathing Instruction: Corpse Pose.

Week 7:
Sutra II.54
Seated/Reclining Poses/Breathing Instruction: (1) Cross-Legged Easy Pose with Upward Hand Pose ×2; (2) *Staff Pose*; (3) Bound Angle Pose.
Sun Salutation: Teach jumping technique. (Step or jump between poses. Use posture sequence 1–18 in Week 1).
Standing Poses: (1) Mountain Pose; (2) *Extended Hand to Big Toe Pose I*; (3) *Extended Hand to Big Toe Pose II*; (4) Intense Side Stretch Pose; (5) Revolved Triangle Pose (use instruction in Week 5).
Back Bends: (1) Standing Back Bend ×3, reach the arms up and back to the wall behind, hold the last one; (2) Supported Supine Back Bend with two blocks; (3) Bridge Pose—Bent Leg Variation; (4) Supported Bridge Pose (with block under sacrum).
Transition Poses/Twists: Bharadvaja's Twist I or *Marichi's Twist III.*
Forward Bends: (1) Child's Pose; (2) *Head to Knee Pose.*
Inversions: Plow Pose to Shoulderstand (use modification in week 4), or Inverted Pose.
Deep Relaxation/Breathing Instruction: Corpse Pose.

(*continued*)

Week 8:
Sutra III.1
Seated/Reclining Poses/Breathing Instruction: (1) Hero Pose; (2) Staff Pose; (3) *Full Boat Pose*; (4) *Half Boat Pose*
Sun Salutation (Step or jump between poses. Use posture sequence 1–18 in Week 1).
Standing Poses: (1) Mountain Pose; (2) *Half Moon Pose*; (3) Warrior I; (4) Warrior III.
Back Bends: (1) *Upward Facing Dog Pose*; (2) Camel Pose; (3) Supported Bridge Pose (with bolster and block against the wall).
Transition Poses/Twists: Marichi's Twist I.
Forward Bends: Child's Pose.
Inversions: Plow Pose to Shoulderstand (use modification in week 4), or Inverted Pose.
Deep Relaxation/Breathing Instruction: Corpse Pose.

Week 9:
Sutra III.2
Seated/Reclining Poses/Breathing Instruction: (1) Hero Pose with Upward Hand Pose ×2, Upward Bound Knuckle Pose ×2, and *Cow Face Pose (Arms)*; (2) Bound Angle Pose; (3) Seated Forward Bend to Plow Pose Cycle (use breath instruction in Week 1).
Sun Salutation (Step or jump between poses. Use posture sequence 1–18 in Week 1).
Standing Poses: (1) Mountain Pose; (2) Triangle Pose; (3) Side Angle Pose.
Back Bends: (1) Standing Back Bend; (2) Supine Bound Angle Pose (Back Bend with blankets in cross, two folded and stacked blankets under the spine and one across for the head); (3) Bridge Pose—Bent Leg Variation; (4) Supported Bridge Pose (with block under sacrum).
Transition Poses/Twists: Half Lord of the Fishes Pose I.
Forward Bend Poses: (1) Seated Wide Angle Pose; (2) *Standing Forward Bend Pose*.
Inversions: Plow Pose to Shoulderstand (use modification in week 4), or Inverted Pose.
Deep Relaxation/Breathing Instruction: Corpse Pose.

Week 10:
Sutra III.3
Seated/Reclining Poses/Breathing Instruction: (1) Cross-Legged Easy Pose; (2) Bound Angle Pose; (3) *Supine Hand to Big Toe Pose I*.
Sun Salutation (Step or jump between poses. Use posture sequence 1–18 in Week 1).
Standing Poses: (1) Mountain Pose; (2) Intense Side Stretch Pose; (3) Revolved Triangle Pose.
Back Bends: (1) Supine Bound Angle Pose (Back Bend with blankets in cross, two folded and stacked blankets under the spine and one across for the Head); (2) Supported Bridge Pose (with bolster and block against the wall).
Transition Poses/Twists: Marichi's Twist III.
Forward Bend Poses: (1) Child's Pose; (2) *Head to Knee Pose*.
Inversions: Plow Pose to Shoulderstand (use modification in week 4), or Inverted Pose.
Deep Relaxation/Breathing Instruction: Corpse Pose.

Weeks 11 and 12:
Sutra I.2
Seated/Reclining Poses/Breathing Instruction: (1) Cross-Legged Easy Pose with Upward Hand Pose ×3 and Upward Bound Knuckle Pose ×2. (2) Seated Forward Bend to Plow Pose Cycle, ×8 with bent knees, ×8 with straight legs (use breath instruction in Week 1).
Sun Salutation ×3: (Step or jump between poses. Use posture sequence 1–18 in Week 1).
Standing Poses: (1) Tree Pose ×2 each side; (2) Warrior II Pose ×2 each side; (3) Triangle Pose ×2 each side; (4) Half Moon Pose ×2 each side; (5) Revolved Triangle Pose ×2 each side.
Back Bends: (1) Standing Backbend ×3; (2) Supported Supine Back Bend; (3) Bridge Pose Bent Leg Variation ×4; (4) Camel Pose ×3; (5) Child's Pose; (6) Supported Bridge Pose (with bolster and block against the wall).
Transition Poses: Supine Twist—Bent Knees Variation ×2 each side.
Forward Bends: (1) Head to Knee Pose; (2) Seated Forward Bend Pose.
Inversions: Plow Pose to Shoulderstand (use modification in week 4), or Inverted Pose.
Transition Poses/Twists: Supine Twist—Bent Knees Variation ×2 each side.
Deep Relaxation/Breathing Instruction: Corpse Pose.

Supplementary Table 2. English and Sanskrit Names of Postures Listed By Intervention Week

All Sanskrit names and posture descriptions are taken from Iyengar BKS: *Yoga The Path to Holistic Health* (Dorling Kindersley, 2008), except those listed below with an asterisk (*) following the Sanskrit name, which are taken from Mehta S, Mehta M, Mehta S: *Yoga The Iyengar Way* (Dorling Kindersley, 1990). The commonly used English names of the following poses are used: Adho Mukha Virasana or Virasana Forward Bend is referred to as Child's Pose, Prasarita Padottanasana or Intense Leg Stretch is referred to as Wide Legged Standing Forward Bend, Paschimottanasana or Intense Back Stretch is referred to as Seated Forward Bend, Tadasana Paschima Namaskarasana, or Mountain Pose with Hands Folded Behind the Back is referred to as Reverse Hand Salute, Tadasana Urdhva Baddhanguliasana, or Mountain Pose with Fingers Interlocked is referred to as Upward Bound Knuckle Pose—Standing, Utthita Parsvakonasana or Extended Side Angle Pose is referred to as Side Angle Pose, and Utthita Trikonasana or Extended Triangle Pose is referred to as Triangle Pose.

English name of poses	*Sanskrit name of Asanas*	*Weeks*										
		1	*2*	*3*	*4*	*5*	*6*	*7*	*8*	*9*	*10*	*11*
Seated/Reclining Poses												
Bound Angle	Baddha Konasana*		x	x			x	x		x	x	
Cow Face (Arms)	Gomukhasana (Arms)*				x		x			x		
Full Boat	Paripurna Navasana								x			
Half Boat	Ardha Navasana*								x			
Hero	Virasana			x	x		x		x	x		
Cross-Legged Easy	Sukhasana*	x	x	x		x		x			x	x
Seated Forward Bend to Plow Cycle	Paschimottanasana/Halasana Vinyasa	x		x			x			x		x
Staff	Dandasana							x	x			
Supine Bound Angle	Supta Baddha Konasana			x								
Supine Hand to Big Toe I	Supta Padangusthasana I*	x			x	x					x	
Supine Hand to Big Toe II	Supta Padangusthasana II*	x			x							
Upward Bound Knuckle -Seated	Parvatasana*	x		x		x				x		x
Upward Extended Legs	Urdhva Prasarita Padasana*					x						
Upward Hand - Seated	Urdhva Hastasana Variation*	x		x		x	x	x		x		x
Sun Salutation Variation	Surya Namaskar Variation*											
Mountain	Tadasana	x	x	x	x	x	x	x	x	x	x	x
Upward Hand	Urdhva Hastasana*	x	x	x	x	x	x	x	x	x	x	x
Standing Forward Bend	Uttanasana	x	x	x	x	x	x	x	x	x	x	x
Standing Forward Bend - Head Up	Uttanasana - Head Up	x	x	x	x	x	x	x	x	x	x	x
Downward Facing Dog	Adho Mukha Svanasana	x	x	x	x	x	x	x	x	x	x	x
Four Limb Staff	Caturanga Dandasana*	x	x	x	x	x	x	x	x	x	x	x
Upward Facing Dog	Urdhva Mukha Svanasana*	x	x	x	x	x	x	x	x	x	x	x
Downward Facing Dog	Adho Mukha Svanasana	x	x	x	x	x	x	x	x	x	x	x
Standing Forward Bend- Head Up	Uttanasana - Head Up	x	x	x	x	x	x	x	x	x	x	x
Standing Forward Bend	Uttanasana	x	x	x	x	x	x	x	x	x	x	x
Upward Hand	Urdhva Hastasana*	x	x	x	x	x	x	x	x	x	x	x
Mountain	Tadasana											
Standing Poses												
Chair	Utkatasana*	x					x					
Extended Hand to Big Toe I	Utthita Hasta Padangusthasana I*							x				
Extended Hand to Big Toe II	Utthita Hasta Padangusthasana II*							x				
Wide Legged Standing Forward Bend	Prasarita Padottanasana		x	x								
Side Angle	Utthita Parsvakonasana		x							x		
Half Moon	Ardha Chandrasana			x					x			x
Intense Side Stretch	Parsvottanasana				x		x	x			x	
Mountain	Tadasana	x	x	x	x	x	x	x	x	x	x	x
Reverse Hand Salute	Tadasana Paschima Namaskarasana				x		x					
Revolved Triangle	Parivrtta Trikonasana*					x		x			x	x
Tree	Vrksasana*	x		x								x
Triangle	Utthita Trikonasana	x		x	x	x			x	x		x
Upward Bound Knuckle - Standing	Tadasana Urdhva Baddhanguliasana		x									
Upward Hand	Urdhva Hastasana*		x				x					
Warrior I	Virabhadrasana I				x		x		x			
Warrior II	Virabhadrasana II	x	x			x						x
Warrior III	Virabhadrasana III*								x			

(*continued*)

Supplementary Table 2. (Continued)

English name of poses	*Sanskrit name of Asanas*	*Weeks*										
		1	*2*	*3*	*4*	*5*	*6*	*7*	*8*	*9*	*10*	*11*
Back Bends												
Setu Bandha Sarvangasana Variation		x	x			x	x		x		x	
Camel	Ustrasana				x	x			x			x
Cobra	Bhujangasana*											x
Standing Back Bend	Urdhva Dhanurasana Prep at Wall*							x		x		x
Supine Bound Angle—Back Bend	Supta Baddha Konasana		x	x			x			x	x	
Supported Bridge—with bolster	Setu Bandha Sarvangasana Variation	x	x				x		x		x	x
Supported Bridge—with block	Setu Bandha Sarvangasana Variation				x			x		x		
Supported Supine Back Bend—with two blocks	Matsyasana Variation*	x		x		x		x				x
Upward Facing Dog	Urdhva Mukha Svanasana*					x			x			
Twists												
Half Lord of the Fishes I	Ardha Matsyendrasana I*				x					x		
Marichi's Twist I	Maricyasana I*		x			x			x			
Marichi's Twist III	Maricyasana III*							x			x	
Bharadvaja's Twist I	Bharadvajasana I*	x					x	x				
Supine Twist—Bent Knees Variation	Jathara Parivartanasana Variation*	x	x									x
Hero with Twist	Parsva Virasana			x								
Forward Bends												
Child's	Adho Mukha Virasana*	x		x	x	x	x	x	x		x	x
Head to Knee	Janu Sirsasana		x		x			x			x	x
Seated Forward Bend	Paschimottanasana		x				x					x
Seated Wide Angle	Upavista Konasana					x				x		
Inversions												
Inverted Pose	Viparita Karani	x	x	x	x	x	x	x	x	x	x	x
Plow	Halasana*				x	x	x	x	x	x	x	x
Shoulderstand	Salamba Sarvangasana*				x	x	x	x	x	x	x	x
Deep Relaxation												
Corpse	Savasana	x	x	x	x	x	x	x	x	x	x	x
